# The Genetic Structures and Molecular Mechanisms Underlying Ear Traits in Maize (*Zea mays* L.)

**DOI:** 10.3390/cells12141900

**Published:** 2023-07-21

**Authors:** Zhenying Dong, Yanbo Wang, Jianxi Bao, Ya’nan Li, Zechao Yin, Yan Long, Xiangyuan Wan

**Affiliations:** 1 Research Institute of Biology and Agriculture, Shunde Innovation School, School of Chemistry and Biological Engineering, University of Science and Technology Beijing, Beijing 100083, China; zydong@ustb.edu.cn (Z.D.);; 2 Beijing Engineering Laboratory of Main Crop Bio-Tech Breeding, Beijing International Science and Technology Cooperation Base of Bio-Tech Breeding, Zhongzhi International Institute of Agricultural Biosciences, Beijing 100192, China

**Keywords:** quantitative trait locus (QTL), quantitative trait nucleotide (QTN), genetic loci, cluster, functional gene

## Abstract

Maize (*Zea mays* L.) is one of the world’s staple food crops. In order to feed the growing world population, improving maize yield is a top priority for breeding programs. Ear traits are important determinants of maize yield, and are mostly quantitatively inherited. To date, many studies relating to the genetic and molecular dissection of ear traits have been performed; therefore, we explored the genetic loci of the ear traits that were previously discovered in the genome-wide association study (GWAS) and quantitative trait locus (QTL) mapping studies, and refined 153 QTL and 85 quantitative trait nucleotide (QTN) clusters. Next, we shortlisted 19 common intervals (CIs) that can be detected simultaneously by both QTL mapping and GWAS, and 40 CIs that have pleiotropic effects on ear traits. Further, we predicted the best possible candidate genes from 71 QTL and 25 QTN clusters that could be valuable for maize yield improvement.

## 1. Introduction

Maize is one of the most important agricultural crops for humans and is widely consumed as food, feed, biofuel, and raw material. Although it has been ranked first in terms of yield per unit and total kernel production [1], there is a continuous increase in demand for maize worldwide, so the enhancement of yield is still one of the main breeding goals [2,3].

**Figure 1:**
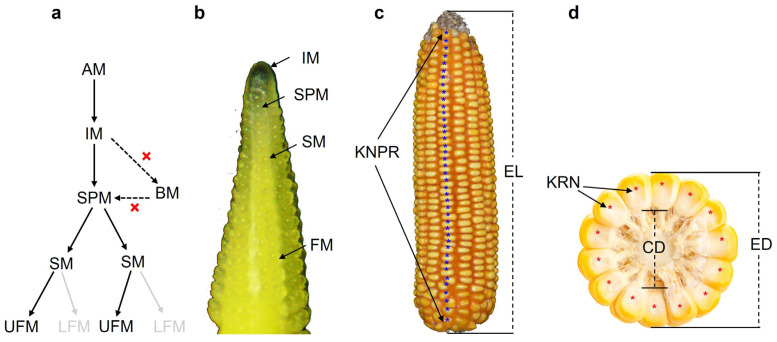
A diagram of the female inflorescence developmental process and ear traits in maize. (**a**) A diagram of female inflorescence developmental process, where the initiation of BMs is inhibited and the LFM of each ear spikelet is aborted during female IM development. (**b**) The different meristem types shown in a developing immature ear. (**c**,**d**) A mature ear and a diagram of the important components of ear traits: AM, axillary meristem; BM, branch meristem; CD, cob diameter; ED, ear diameter; EL, ear length; EN, ear number; IM, inflorescence meristem; KNPR, kernel number per row; KRN, kernel row number; LFM, lower floret meristem; SM, spikelet meristem; SPM, spikelet pair meristem; UFM, upper floret meristem.

Maize is a monoecious plant that develops unisexual male and female flowers in physically separated parts of the plant [4]. The male inflorescence, the tassel, directly derives from the shoot apical meristem (SAM) formed during embryogenesis, while the female inflorescences derive from axillary meristems (AM) soon after germination in the axils of leaves [5,6]. The female inflorescence development begins after the conversion of AM into the inflorescence meristem (IM). The IM initiates spikelet pair meristems (SPMs) initially, and each SPM quickly generates a pair of spikelet meristems (SMs). Subsequently, each SM forms a pair of floral meristems (FMs), and the process of IM to branch meristems (BMs) that occurs in the base of male IM is inhibited during female IM differentiation (Figure 1a). The upper floret meristem (UFM) develops fully, but the lower floret meristem (LFM) of each ear spikelet is aborted early in its development, resulting in two mature kernels being formed from one SPM (Figure 1a). The apexes of both male and female inflorescences initiate primordia successively, which mature gradually as they are displaced towards the base; thus, different developmental stages of meristems from the youngest at the apex to the oldest at the base can be observed during maize inflorescence development (Figure 1b). Elongation and the ability to differentiate female IM during maize development are closely related to the final ear traits and kernel yield [7,8]. Although maize plant initiates a series of AMs in the axils of leaves during development, only the highest axillary buds can transit to form ears in most cases, and modern commercial maize varieties have only one or two ears per plant. For the mature maize ears, ear number (EN) or prolificacy, ear length (EL), ear diameter (ED), ear weight (EW), kernel row number (KRN), kernel number per row (KNPR), kernel weight per ear (KWPE), cob diameter (CD), cob weight (CW), etc., constitute the main components of ear traits (Figure 1c,d).

Both molecular and archaeobotanical evidence have shown that maize was domesticated from teosinte (*Zea mays* subsp. parviglumis) in Mexico about 9000 years ago [9,10,11], but the plant morphology is significantly different from its ancestor. Teosinte has multiple long lateral branches and produces several small ears at multiple nodes along these lateral branches, resulting in tens of ears per teosinte plant, but the ear is tiny, with only two rows and about 5–12 kernels [12,13,14]. After domestication, an increase in apical dominance in maize resulted in only one main stalk or very few lateral branches, as well as lower prolificacy. However, the significantly enlarged maize ear can bear hundreds of kernels without a concomitant loss in yield per plant [10,12,13,14].

Maize yield is determined by multiple components, including ear number per unit land area, KNPR, and KW. Under a certain plant density, kernel number is far more important and responsible for most of the yield variation [15,16]. The majority of the ear characteristics, such as EN, EL, EW, KRN, and KNR, have a significant role in determining the final maize yield. Therefore, the dissection of the genetic structures underlying ear traits and the utilization of the elite alleles are vital for enhancing maize yield potential. The components of maize yield, including ear traits, are typical polygenic traits that are controlled by quantitative trait loci (QTLs), and quantitative genetic approaches, including QTL mapping and genome-wide association study (GWAS), have been widely applied for decoding their genetic basis, and numerous QTLs and quantitative trait nucleotides (QTNs) regulating the ear traits have been detected [17,18]. Additionally, a number of mutants affecting female inflorescence development and ear traits have been identified, which further deepens the understanding of molecular regulation of maize ear traits [19,20]. The application of such forward and reverse genetic approaches has provided mutually complementary evidence for the genetic regulatory mechanisms of ear traits. The mutants having positional information on the causal genes provide candidate genes for ear architecture QTLs; conversely, when a QTL maps to a region without known genes, it provides positional information for new gene discovery [21]. Furthermore, if a chromosomal position was repeatedly revealed by QTL mapping, GWAS, or both types of research, the function of such a genetic locus should be environmentally stable, and it is more useful for maize breeding [22].

Many studies have summarized the molecular regulation of grass inflorescence meristem development [20,23,24], the specific developmental stages of cereal crops [25], and the quantitative loci regulating maize inflorescence architecture [18,22]. However, the genetic and molecular networks of the ear traits underlying maize yield have not been summarized, which impedes the utilization of such knowledge for enhancing maize yield. Here, we simultaneously refine the environmentally stable genetic loci, as well as the pleiotropic loci regulating multiple ear traits, by collecting the ear-trait-related QTL and QTN data, and we construct the molecular networks underlying ear traits by collecting information on known ear-trait-related genes. In addition, we predict new candidate genes regulating ear traits, which will be useful for further revealing the molecular regulatory mechanisms of ear traits and maize yield improvement.

## 2. Materials and Methods

### 2.1. The Literature Review and QTL/QTN Data Collection

A systematic literature search was conducted against the Web of Science (https://www.webofscience.com/, accessed on 1 January 2023) and PubMed (https://pubmed.ncbi.nlm.nih.gov/, accessed on 1 January 2023) databases using the search terms [(TS = (maize OR “Zea mays”)) AND (TS = (GWAS OR “genome wide association study” OR QTL OR “quantitative trait loci” OR “gene”)) AND TS = ear trait]. To refine the maize QTLs/QTNs responsible for ear traits, including EN, EL, ED, EW, KRN, etc., a total of 70 independent studies focusing on ear trait loci identification (44 for QTL, 21 for QTN, and 5 for both), which represent all the studies over the past 20 years, were retrieved and used for further analysis after removing unrelated articles. In addition, 56 articles (with 1 article that overlaps with the 70 studies mentioned above) on 48 functional genes regulating ear traits were also identified.

### 2.2. The Construction of a Consensus Map for Ear Traits

QTLs and QTNs were obtained from the above-mentioned 70 articles. However, due to differences in marker types, genomic mapping methods, and reference genome versions, the genetic location information cannot be unified. Therefore, sequences of the flanking markers of QTLs were searched by applying the BLAST program on the maize GDB database (https://www.maizegdb.org, accessed on 2 March 2023) and normalized to the B73 reference genome (RefGen_v5.0). For the QTNs, all the positional information was transformed to the same B73 version 5 by using the version conversion tool in NCBI (https://www.ncbi.nlm.nih.gov/genome/tools/remap, accessed on 2 March 2023).

### 2.3. The Identification of QTN and QTL Clusters

To identify the localization hotspots of QTLs and QTNs, we defined the QTL region that could be mapped by three or more independent studies as a QTL cluster, but if the interval of a QTL cluster is more than 30 mega-base pairs (Mb), due to more QTLs overlapping each other, five or more independent studies were set as a new criterion. For QTNs, the regions containing three or more associated QTNs for each trait in a 0.5 Mb sliding window were defined as a QTN cluster; this criterion is relatively stricter than the previous report on defining male inflorescence QTN clusters [22].

### 2.4. The Construction of the Molecular Networks Regulating Ear Traits and Candidate Gene Identification of QTN and QTL Clusters

The 48 known genes regulating ear traits were retrieved from a literature review. Depending on the description of the genetic or protein–protein interactions and transcriptional or post-transcriptional regulatory models for specific genes in the literature, the molecular regulatory networks for female inflorescence development and ear traits were proposed by combining all the above data. To identify the candidate genes of QTN and QTL clusters, we first extracted the female IM-expressed genes [26], and then checked the annotation of the genes located in the QTL or QTN clusters. If the genes belonged to the summarized networks or were annotated as the same types of known genes, they were assigned as the most possible candidate genes.

### 2.5. Abbreviations

A list of the 99 abbreviations used in this study is provided in Table S1.

## 3. Results and Discussion

### 3.1. The Genetic Basis of the Ear Traits and Summary of the Genetic Loci Identified by QTL Mapping

Grain yield is one of the most important traits for maize production, but the low heritability and the fact that it is easily influenced by the environment result in its low breeding efficiency [27]. However, many yield components, including ear and kernel traits, which also significantly correlate with grain yield, show higher heritability and better stability across different environments [27,28], making yield components more suitable to be employed to facilitate maize breeding.

A literature review revealed that QTL mapping for ear traits has emerged ever since the application of molecular markers. For example, by using the F_2:3_ lines and RFLP markers, Veldboom and Lee [28] identified 14 genomic regions associated with yield component traits including CD, ED, EL, and KRN, and some of the loci are located in the previously reported regions that were detected by isozyme markers [29]. Upadyayula et al. [21] detected 18 QTLs for ear traits from 150 S_1_ families derived from (ILP × B73) B73, and pleiotropic loci regulating multiple traits were also potentially identified, some of which locate in regions with known genes that affect ear development. The authors also found that only a few QTLs overlap with previously reported loci, and they proposed that this may be due to different parents being used for the construction of the mapping population, and epistasis might modulate the effect of a QTL depending on the genetic background [21]. KRN is one of the most important ear trait components and an important selection target during maize domestication and breeding [13,14,30,31]. Many efforts have been made to dissect the genetic basis of KRN and more than three hundred QTLs have been identified [13,30,31,32,33,34], and at least four of the KRN QTLs have been cloned [35,36,37,38]. A significant amount of information on the genetic basis for the most important ear traits has accumulated, which provides a solid basis for combining the different individual studies to summarize the genetic regulation of ear traits. Based on this consideration, we initially collected QTLs from published QTL mapping articles [16,21,30,31,32,33,34,39,40,41,42,43,44,45,46,47,48,49,50,51,52,53,54,55,56,57,58,59,60,61,62,63,64,65,66,67,68,69,70,71,72,73,74,75,76,77,78,79,80,81,82,83,84,85,86,87,88,89,90]. A consensus map for ear trait QTLs was constructed with the positional information of all the original QTLs, which was normalized to the same version of B73 reference genome (RefGen_v5.0) when the primer sequences of the molecular markers were available.

**Figure 2:**
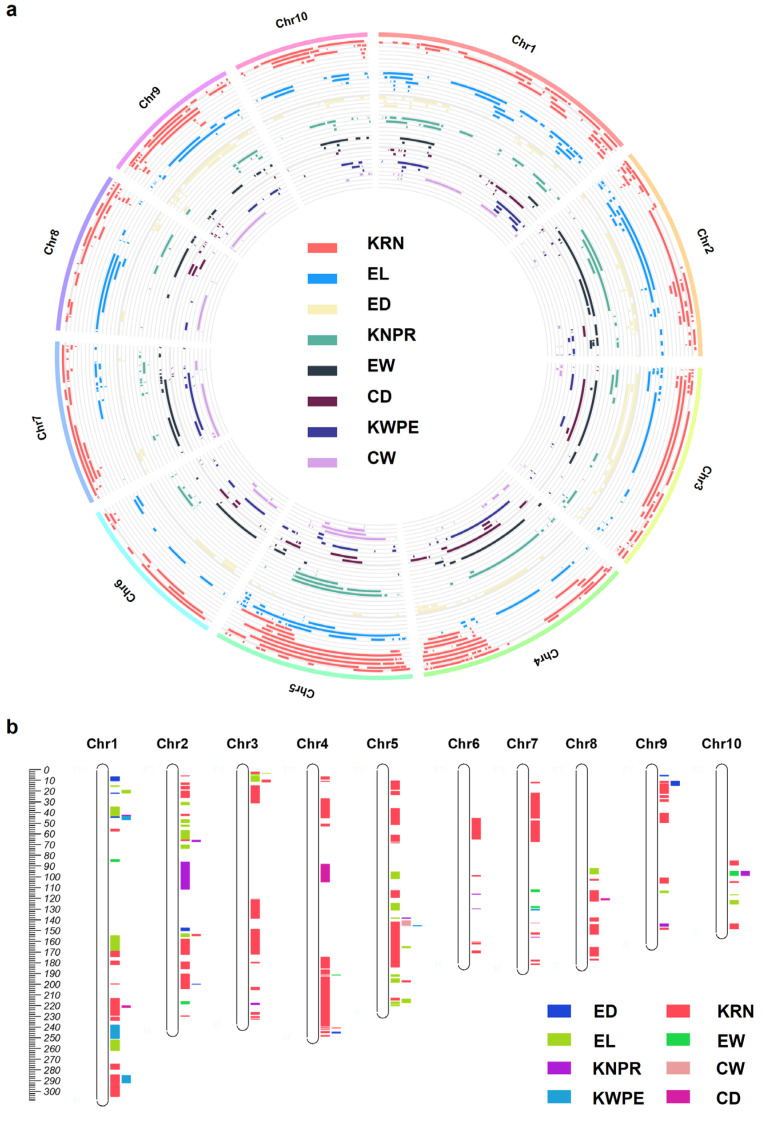
A summary of the ear-trait-related QTLs (**a**) and chromosomal locations of the QTL clusters for ear traits in maize (**b**). CD, cob diameter; CW, cob weight; ED, ear diameter; EW, ear weight; EL, ear length; KRN, kernel row number; KNPR, kernel number per row; KWPE, kernel weight per ear.

After normalization, we collected 328 KRN, 180 EL, 134 ED, 113 KNPR, 84 EW, 75 CD, 74 KWPE, and 68 CW QTLs (Figure 2a, Table S2). Subsequently, we analyzed the data of these eight ear traits in this study considering their importance on maize yield. The ear trait QTLs were detected on all 10 maize chromosomes, and chromosome 1 (Chr1) to Chr10 possessed 160, 141, 115, 122, 114, 53, 101, 81, 100, and 69 QTLs, respectively (Figure 2a). To further reveal the QTLs for specific traits that can be repeatedly detected by different independent research groups or from different environments, we defined the QTL cluster as described (see Section 2). In total, 153 including 90 KRN, 28 EL, 8 ED, 10 KNPR, 6 EW, 3 CD, 5 KWPE, and 3 CW QTL clusters were refined (Figure 2b, Table S3). The intervals of the QTL clusters range from 0.02 to 43.9 Mb, with 45 clusters shorter than 1 Mb and 133 clusters shorter than 10 Mb (Figure 2b, Table S3). Furthermore, no QTL cluster is distributed on the short arms of Chr8 and Chr10 (Figure 2b, Table S3).

### 3.2. The Genetic Basis of the Ear Traits and Summary of the Genetic Loci Identified by GWAS

Due to the fast development of genotyping technologies and wide genetic diversity within the population, high-throughput single-nucleotide polymorphism (SNP) markers are easily available, and the linkage disequilibrium (LD) decays rapidly in the maize population, making it an ideal crop for GWAS [91]. GWAS has been applied in dissecting the genetic basis of many important agronomic traits, including the male and female inflorescence traits of maize [18,22].

A relatively early study on the genetic architecture dissection of ear traits by GWAS was reported by Brown et al. [17], which measured cob length (CL), CD, and KRN in the maize nested association mapping (NAM) population, including 4892 recombinant inbred lines (RILs), and identified 233, 317, and 261 significantly associated QTNs, respectively. They also performed joint linkage mapping and identified 26, 39, and 36 QTLs, respectively [17]. It was further found that only a small degree of overlap existed between the locations of associated QTNs and the known genes that were cloned by mutant screening, and they proposed that it is possibly caused by a lower efficiency of the mutant screening on detecting gene redundancy [17]. An improved new nonparametric model, called the Anderson–Darling (A–D) test, was combined with a mixed linear model (MLM) to reveal the genetic architecture of 17 agronomic traits, including EL, ED, CD, KNPR, and CW, and the results showed that some known loci and new candidate loci were only observed by the A–D test [92]. By using multiple populations, Xiao et al. [93] found 17–34 minor- to moderate-effect loci accounting for 55.4–82% of the total phenotypic variation in ear traits. More recently, 51 environmentally stable and 36 pleiotropic QTNs regulating the ear traits, including CD, CW, ED, EL, EW, and KRN, were identified from an association panel comprising 362 inbred lines [34]. By comparison, only 16 environmentally stable and 16 pleiotropic QTLs were identified by linkage mapping [34].

**Figure 3:**
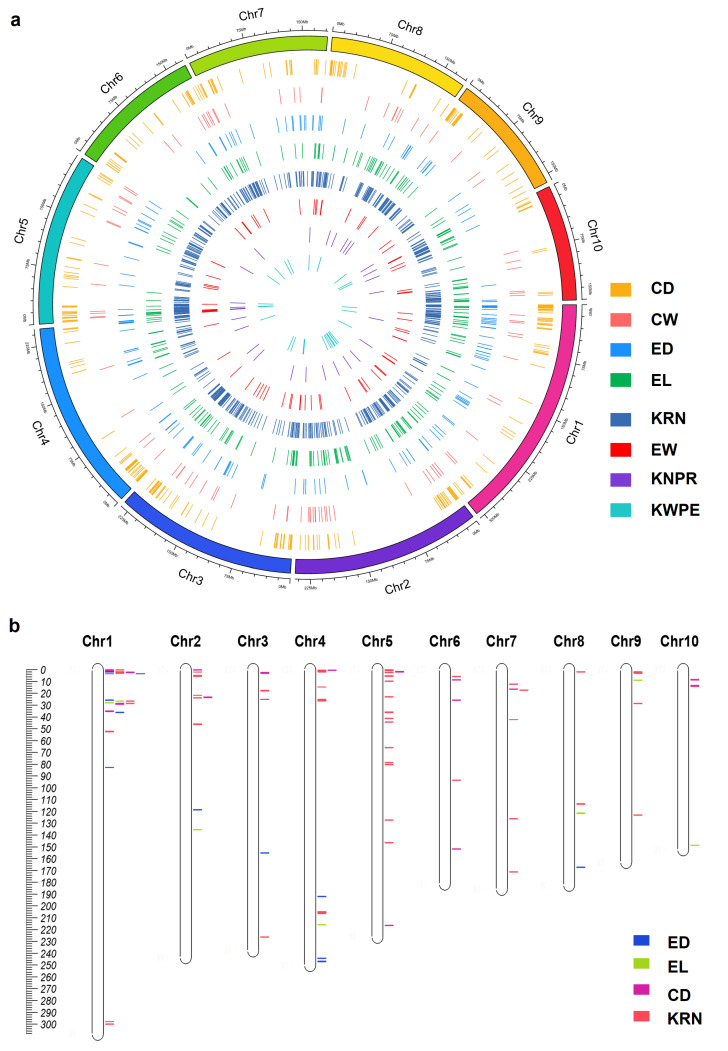
A summary of the ear-trait-related QTNs (**a**) and chromosomal locations of the QTN clusters for ear traits in maize (**b**). CD, cob diameter; CW, cob weight; ED, ear diameter; EW, ear weight; EL, ear length; KRN, kernel row number; KNPR, kernel number per row; KWPE, kernel weight per ear.

To further uncover the genetic regulation of ear traits revealed by GWAS research, we collected the known QTNs associated with ear traits from published papers [17,33,34,35,77,80,87,92,93,94,95,96,97,98,99,100,101,102,103,104,105,106,107,108,109,110]. Similar to the normalization of QTLs, 829 KRN, 377 CD, 271 EL, 183 ED, 98 CW, 78 EW, 29 KNPR, and 28 KWPE QTNs were successfully mapped to B73 RefGen_v5.0 (Figure 3a, Table S4). Ear trait QTNs were detected in all 10 maize chromosomes, and Chr1 to Chr10 possess 314, 241, 201, 202, 270, 131, 137, 141, 138, and 118 QTNs, respectively (Figure 3a).

We think the important regions regulating ear traits should be enriched by significantly associated QTNs; thus, the regions containing three or more associated QTNs for each trait in a 0.5 Mb sliding window were defined as a QTN cluster. With this criterion, 85 including 48 KRN, 7 EL, 11 ED, and 19 CD, but not CW, EW, KNPR, or KWPE QTN clusters were refined (Figure 3b, Table S5). The intervals of the QTN clusters ranged from 0.5 to 1.53 Mb, with 82 clusters shorter than 1 Mb (Figure 3b, Table S5). Ear trait QTN clusters were detected on all 10 maize chromosomes, and Chr1 to Chr10 possess 21, 9, 5, 12, 16, 5, 6, 4, 4, and 3 QTN clusters, respectively (Figure 3b, Table S5). Although Chr1 possesses the largest number of QTN clusters, most of the clusters are located in the proximal region of the short arm, and similar patterns also occur in other chromosomes, except for Chr4 and Chr8 (Figure 3b, Table S5). Although the number of QTNs is larger than the QTLs, the number of QTN clusters is less than the QTL clusters, and they are not evenly distributed on the chromosomes (Figure 3). We think this is potentially caused by the use of a relatively strict standard to define QTN clusters in this study.

### 3.3. A summary of the Molecular Mechanisms Regulating Ear Traits Based on Information of the Known Genes, and the Instruction for Prediction of the Candidate Genes of QTL and QTN Clusters

**Figure 4:**
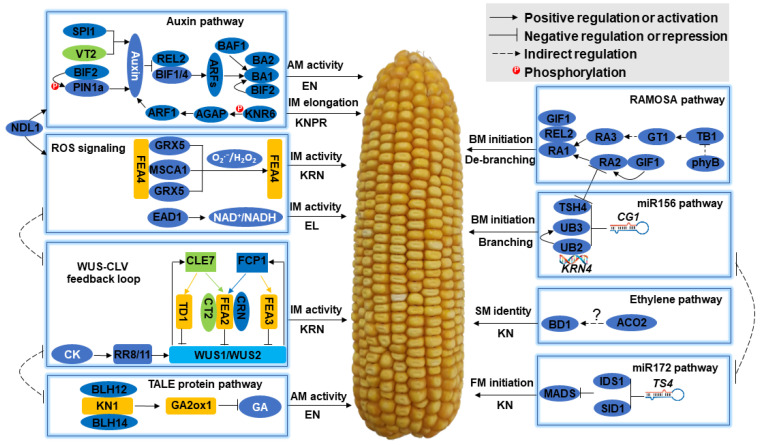
Molecular mechanisms regulating female inflorescence development and ear traits. Eight modules are summarized, and the function of each module on female inflorescence development and ear traits are indicated: AM, axillary meristem; BM, branch meristem; CD, cob diameter; CK, cytokinin; CLV, CLAVATA; ED, ear diameter; EL, ear length; EN, ear number; FM, floral meristem; GA, gibberellin; IM, inflorescence meristem; KN, kernel number; KNPR, kernel number per row; KRN, kernel row number; miR156, microRNA156; miR172, microRNA172; ROS, reactive oxygen species; SM, spikelet meristem; TALE, three amino acid loop extension; WUS, WUSCHEL.

From the initiation of AMs in the axils of leaves where female IM will be formed, to the differentiation of FMs that will form kernels ultimately, any disturbance or gene mutation will alter ear traits and affect grain yield. Many efforts have been made to identify the genes underlying ear traits by using both forward and reverse genetic methods, and more than 40 genes regulating female inflorescence development and ear traits have been cloned; however, the regulatory mechanisms remain largely unknown. By summarizing and analyzing the original papers, we constructed a molecular network regulating ear traits (Figure 4), and all the detailed information for the genes is provided in Table S6.

#### 3.3.1. The Auxin Pathway and Regulation of AM Activity and Lateral Primordia Initiation

Auxin plays a central regulatory role in AM initiation and subsequent lateral primordia initiation in IM. As a result, most mutants relating to auxin pathway genes have reduced ear numbers or display pin-like inflorescences, which are classically called *barren* mutants [111].

Mutations of any genes regulating auxin biosynthesis, transportation, or singling may have serious effects on ear development. *SPARSE INFLORESCENCE1* (*SPI1*) encodes a monocot-specific YUCCA-like gene that participates in auxin biosynthesis and regulates the formation of Ams; the *spi1* mutant has a significantly reduced number of AMs and female IMs are fasciated with fewer SPMs so that the mature ears show a reduced kernel number [112]. *VANISHING TASSEL2* (*VT2*) is an ortholog of *TRYPTOPHAN AMINOTRANSFERASE OF ARABIDOPSIS1* (*TAA1*) that functions in Trp-dependent auxin biosynthesis. VT2 regulates both vegetative and reproductive development and the *vt2* mutant is much shorter and has fewer ears than the WT (wild type). At maturity, the ears show an obvious decrease in length and kernel number, and *spi1/vt2* double mutants resemble *vt2*, but EL and KWPE are further reduced [113].

Auxin transport is directed by the PIN-FORMED (PIN) family of auxin efflux carriers, and serine/threonine protein kinase PINOID (PID) regulates the subcellular localization of PIN proteins [114]. In maize, *BARREN INFLORESCENCE2* (BIF2) is co-orthologous to *PID* serine/threonine kinase [115], and the *bif2* mutant fails to produce ear shoots or initiate branch meristem florets, resulting in a reduced number of ears with fewer kernels [116]. Maize has three genes orthologous to *AtPIN1*, designated as *ZmPIN1a*, *ZmPIN1b,* and *ZmPIN1c* [117,118]. Similarly, in *Arabidopsis*, BIF2 can phosphorylate and regulate the subcellular localization of ZmPIN1a [116]. Under-driven by the barley *Pht1* promoter, *ZmPIN1a* overexpression lines were generated and it was found that these lines had more ears and a higher grain yield than WT at different cultivation densities, indicating that *ZmPIN1a* are valuable in maize breeding [119].

After transportation to the target sites, auxin binds to the receptor TRANSPORT INHIBITOR RESPONSE1 (TIR1) or AUXIN-related F-BOX (AFB) proteins, which facilitate the degradation of AUXIN/INDOLE-3-ACETIC ACID (AUX/IAA) repressors and disrupt the recruitment of TOPLESS (TPL) corepressors, and as a result, AUXIN RESPONSE FACTOR (ARF) transcription factors (TFs) are released from the repressor complex and activate downstream genes [120]. In maize, *BIF1* and *BIF4* encode AUX/IAA proteins, the semi-dominant *Bif1* and *Bif4* mutants have severe defects in the primordia initiation of the IMs, and the ears are significantly shortened with reduced and disorganized rows of kernels [111]. BIF1 and BIF4 can physically interact with RAMOSA ENHANCER LOCUS2 (REL2), a functional homolog of TPL and multiple ARF numbers in maize, and then various activating ARFs can regulate the target gene *BARREN STALK1* (*BA1*), which encodes a basic helix–loop–helix (bHLH) TF [111]. The *ba1* is the first discovered *barren* mutant without reproductive AMs and shows an earless phenotype [121]. Further analysis has shown that BA1 is regulated by multiple components, and BIF2 can also interact with, and phosphorylate, BA1 [122]. *BARREN STALK FASTIGIATE1* (*BAF1*) encodes an AT hook TF, and AMs of the *baf1* mutants either fuse to the culm and generate shorter ears with unorganized rows of kernels or fail to initiate and show a completely earless phenotype. Further double mutant and expression analysis has shown that BAF1 is required for *BA1* expression [123]. *BA2* encodes a protein that heterodimerizes with BA1 in the nucleus [124]. The phenotype of *ba2* mutant is similar to *ba1* in that it fails to produce ears; however, a double-mutant analysis has shown that BA2 works in parallel with BAF1 and BIF2 during AM development [124].

*KERNEL NUMBER PER ROW6* (*KNR6*) encodes a serine/threonine protein kinase and positively regulates the elongation of IM and the ability of floret production [37]. The *KNR6* RNAi lines have shorter ears, whereas the overexpressing lines produce longer ears with more kernels per row, indicating that *KNR6* positively regulates ear-yield-related traits. Furthermore, KNR6 can interact with and phosphorylate an Arf GTPase-activating protein (AGAP) [37]. AGAP also has positive regulatory roles in IM activity, and ear growth is obviously inhibited in *agap* knockout lines [125]. A protein interaction assay showed that AGAP interacts with Arf GTPase1 (ARF1) members. As AGAP and ARF1 interfere with the auxin influx [126], KNR6–AGAP–ARF1 complexes are considered to participate in ear trait regulation by auxin transport mediated by the intracellular trafficking system in maize [125].

#### 3.3.2. Three-Amino-Acid Loop Extension (TALE) Homeodomain Proteins and the Regulation of AM Activity and EN

TALE homeodomain proteins, including Class I KNOTTED1-like homeobox (KNOX) TFs, play important roles in maintaining meristematic cell identity during plant reproductive development [127,128,129,130]. Recessive mutation of maize *KNOX* gene *KNOTTED1* (*KN1*) causes severe inflorescence and floral defects [131,132]. AM initiation is affected in *kn1* and the ears are either absent or small with a poor seed set [131,132,133]. KN1 positively regulates *GA2ox1*, which codes an enzyme that inactivates gibberellin (GA) and thus negatively regulates the accumulation of GA [134]. AM regulation by GA has been reported in other plants [135,136], but whether and how the GA pathway regulates maize AM and ear development must be further studied.

BELL1-like homeobox (BLH) TFs are another type of TALE protein, and they interact with KNOX proteins to produce functional heterodimers [137]. In maize, BLH12 and BLH14 are homologs of *Arabidopsis* PENNYWISE, which interacts with KNOX protein SHOOTMERISTEMLESS [138,139]. Protein interaction assays have shown that both BLH12 and BLH14 interact with KN1, and single mutants of *BLH12* or *BLH14* develop relatively normally, but *blh12/14* double mutants show defects in AM initiation and maintenance, which results in an earless phenotype, indicating that *BLH12* or *BLH14* work redundantly [140].

#### 3.3.3. The CLAVATA (CLV)-WUSCHEL (WUS) Negative Feedback Loop and Regulation of IM Activity and KRN

After AM initiation, the IM proliferation and differentiation in generating a series of spikelet and floral meristems determine the ear size and kernel number per ear and the yield at the mature stage [15,16]. In plants, IM activity is mainly regulated by the evolutionally conserved CLV-WUS negative feedback signaling [20,141,142]. *WUS* encodes a homeodomain containing TF and triggers stem cell proliferation, and its mutation results in the ectopic initiation of shoot meristems and premature termination of floral meristems [141]. In the CLV signaling pathway, *CLV1* encodes a receptor kinase and *CLV2* encodes a receptor-like protein, while *CLV3* encodes a small peptide ligand [143,144,145]. The secretion of CLV3 is perceived by CLV1 or CLV2, and then the signal is transmitted to suppress the expression of WUS [144,146,147]. In turn, *WUS* expression induces the expression of CLV3 [141]. Mutations of the CLV components result in increased IM size, as well as increased numbers of floral organs [141,148].

In maize, the THICK TASSEL DWARF1 (TD1) is the putative ortholog of the *Arabidopsis* CLV1, the female IM of *td1* mutant is fasciated and bifurcated accompanying the formation of supernumerary and larger SPMs that produce spikelet triplet clusters, and the mature ear is fasciated with extra rows of kernels, indicating that TD1 functions to limit meristem size during inflorescence development [149]. FASCIATED EAR2 (FEA2) and FEA3 are maize orthologs of CLV2, and CLV3/EMBRYO-SURROUNDING REGION (CLE) peptide ZmCLE7 and ZmFON2-LIKE CLE PROTEIN1 (ZmFCP1) are CLV3 orthologs [150,151]. FEA2 can respond to ZmFCP1 and ZmCLE7 through two downstream effectors CORYNE (CRN) pseudokinase (ZmCRN) and the α subunit of the G protein COMPACT PLANT2 (CT2) [152,153,154]. FEA2 physically interacts with ZmCRN and CT2, but ZmCRN and CT2 function specifically in ZmFCP1 and ZmCLE7, respectively [152,154]. However, downstream effectors for FEA3 and TD1 have not been reported. FEA3 perceives the ZmFCP1 peptide specifically and then represses the expression of *ZmWUS1* [151]. Like *td1*, all the other loss-of-function mutants of participants in the CLV signaling pathway, including *fea2*, *fea3*, *Zmfcp1*, *Zmcrn*, and *ct2*, show similar enlarged and fasciated ears and extra and irregular kernel rows, which increase KRN [150,151,152,153,154]. In addition, *ct2* forms more female inflorescences on the shanks, which increases prolificacy [155].

Two *WUS* orthologs, *ZmWUS1* and *ZmWUS2,* were identified in the maize genome [156]. *Barren inflorescence3* (*Bif3*) is a dominant mutant harboring a tandem duplicated copy of the *ZmWUS1*, with ectopic and over-expressed *ZmWUS1*. *Bif3* mutant shows enlarged IMs and a reduced number of SPMs, and the mature ears are short and ball-shaped, carrying very few kernels [157]. Two type-A ZmRR proteins in the cytokinin (CK) pathway, ZmRR8 and ZmRR11, can bind to the *ZmWUS1* promoter and activate its transcription, indicating that CK can induce WUS expression in maize, which is similar to that in *Arabidopsis* [157,158]. CK is critical for meristem maintenance and it acts antagonistically to GA [159]. Identification of the TALE protein regulatory network in maize meristems has shown that KN1 could directly target *GA2ox1*, which participates in the GA pathway, and genes in the CK and auxin pathways [133,134]. Thus, it is proposed that the CLV-WUS feedback loop should have some correlations with the TALE protein pathway via CK-GA interaction in the regulation of traits of EN and KRN.

#### 3.3.4. Reactive Oxygen Species (ROS) Signaling and Regulation of IM Activity and KRN

*FEA4* encodes a basic region leucine zipper (bZIP) TF, and it is expressed throughout the entire IMs, SPMs, and SMs in the ear and tassel. The female IMs of the *fea4* mutant are enlarged and fasciated, which resemble other *fea* mutants such as *fea2*, and KRN significantly increases under different genetic backgrounds. However, fea2/fea4 double mutants show significantly larger meristems than both the single mutants, indicating that FEA4 may regulate meristem size by an unknown mechanism in parallel to the CLV pathway [160].

MALE STERILE CONVERTED ANTHER1 (MSCA1) is a glutaredoxin (GRX), which functions redundantly with the paralogues ZmGRX2 and ZmGRX5, with MSCA1 and ZmGRX5 being the major players [161,162]. The *msca1/grx2/grx5* triple mutant has reduced abilities of SPM initiation and SM differentiation, resulting in extremely small ears and much fewer kernels [162]. MSCA1 and the two paralogues can physically interact with FEA4 and promote the reduction in FEA4. The activity and ratio of monomer/dimer of FEA4 is sensitive to the redox state, and under *msca1/grx2/grx5* triple-mutant backgrounds, the oxidized dimeric form of FEA4 increases and enhances DNA accessibility and transcription activity in vivo, indicating that normal ear development needs the proper monomer/dimer balance of FEA4 under regulation by GRXs [162]. As the cellular redox state is closely related to ROS homoeostasis, it is proposed that the three GRX proteins serving as redox regulators participate in ROS signaling by modulating the FEA4 protein activity during early ear development [162]. WUS is also antagonistically regulated by redox signaling in controlling the plant stem cell fate [163], indicating that the components in the ROS signaling pathway may interact with the CLV-WUS pathway for regulating ear traits. In addition, *MSCA1* is a genic male sterility gene, and *mscal* mutation does not affect female fertility [164]. We previously successfully developed multi-control sterility systems (MCSs) using the genic male sterility genes *ZmMs7* and *ZmMs30* for hybrid maize seed production [165,166], and we propose that *MSCA1* also has potential in developing new MCSs.

Malate is an intermediate metabolite in multiple metabolism pathways and plays a role in ROS production and scavenging [167,168,169,170]. *EAR APICAL DEGENERATION1* (EAD1) encodes an aluminum (Al)-activated malate transporter (ALMT), and the *ead1* mutant shows significantly shorter ears, while overexpression of *EAD1* has led to greater EL and KNPR, indicating that malate metabolism plays important roles in female inflorescence development [8]. Further study has shown that the mutation of *EAD1* results in a lower malate content and an increased ratio of NAD+/NADH in the apical part of developing inflorescences, which is considered a typical determinant of cellular redox status [8]. Excessive accumulation of ROS was subsequently observed in apical IM, which further triggers programmed cell death (PCD) and finally results in ear apical degeneration [8]. However, the ROS burst and PCD were only observed until the inflorescences developed to 15 mm in length, indicating that redox can regulate ear development at different stages [8,162,163].

NEEDLE1 (NDL1) encodes an ATP-dependent metalloprotease, defects occur during AM initiation, and IMs occasionally show slight fasciation in *ndl1* ears [171]. Simultaneous observation of strong genetic interactions with auxin pathway genes and the hyperaccumulation of ROS in *ndl1* inflorescences has provided evidence connecting meristem auxin and redox status in the control of maize growth [171].

#### 3.3.5. The RAMOSA (RA) Pathway and Inhibition of Branch Initiation in Maize Ears

During maize reproductive development, the base of the male IM initiates a varying number of BMs and eventually forms long branches at the mature stage, while BM initiation is inhibited during ear development. However, ear branching was observed in some mutants that primarily involve the genes of the RA pathway, including *RA1*, *RA2,* and *RA3* that encode a Cys2-His2 zinc finger protein, a LOB-domain-containing TF, and a trehalose-6-phosphate phosphatase, respectively [172,173,174]. The typical and common phenotypes for the RA pathway genes are the increased indeterminacy of the meristem resulting in the formation of long branches and irregular seed rows in the ear; further double-mutant analysis has shown that RA3 and RA2 act upstream of RA1 [172,174]. Mutation of *REL2* can enhance the phenotypes of *ra1* and *ra2*, even though the *rel2* single mutant does not have obvious phenotypes [175]. REL2 can physically interact with RA1, indicating the REL2/RA1 complex represses the formation of indeterminate branches during maize inflorescence development [175]. Growth-regulating factor (GRF)-interacting factor1 (GIF1) regulates male and female IMs differentially; the *gif1* mutant has extra branches in the ears, but fewer branches in the tassels than WT [176]. Further analysis has shown that GIF1 directly interacts with REL2 and directly binds with and regulates RA2 [177].

The *grassy tillers1* (*gt1*) is a mutant showing increased lateral branches and increased ear numbers, and positional cloning showed that *GT1* encodes a class I homeodomain leucine zipper (class I HD-Zip) gene that suppresses lateral bud outgrowth [178]. RA3 was identified as a GT1 enhancer for the carpel suppression phenotype, while *gt1/ra3* double-mutant analysis showed that *gt1* suppressed the *ra3* tassel and ear branching phenotype, indicating that genetic interaction happened between RA3 and GT1 [179]. *GT1* expression is dependent on the activity of phytochrome B and TEOSINTE BRANCHED1 (TB1), indicating that light plays a role in regulating AM growth in maize [178,180].

TASSELSHEATH4 (TSH4) plays opposite roles with RA pathway genes in regulating ear branching. *TSH4* encodes an SBP-box TF, and the *tsh4* mutant shows reduced BMs in the tassel, and the ear has large bract leaves and spiral kernel rows. Double-mutant and gene expression analysis showed that the RA pathway genes negatively regulate TSH4, and mutual negative regulation of TSH4 with RA2, but not with RA1 and RA3, was also observed [181]. UNBRANCHED2 (UB2) and UB3 are two paralogs of TSH4 and work redundantly to regulate lateral primordia initiation [182]. Together with *TEOSINTE GLUME ARCHITECTURE1* (*TGA1*), a gene that has a role in the domestication of maize from teosinte [183], these SBP-box genes are targets of microRNA156 (miR156) that is encoded by *Corngrass1* (*CG1*) [184]. UB2 and UB3 affect ear length and diameter and the double mutant shows a significant increase in KRN. Interestingly, the UB3 locus is tightly linked to the QTLs for KRN, indicating that this gene is agronomically useful [182].

*KERNEL ROW NUMBER4* (*KRN4*) was identified as a QTL regulating KRN and locates about 60 Kb downstream of UB3 [35]. KRN4 acts as an enhancer and interacts with the *UB3* promoter. Meanwhile, UB2 can directly bind to both *KRN4* and the *UB3* promoter to positively regulate *UB3* expression [185].

#### 3.3.6. The Ethylene Pathway and Regulating IM Activity and SM Identity

The *1-aminocyclopropane-1-carboxylate oxidase2* (*ACO2*) was identified as the causal gene of *qEL7*, a major QTL regulating EL and KNPR, and ACO2 functions in the final step of ethylene biosynthesis and is expressed in developing inflorescences [7,64]. ACO2 affects kernel number (KN) and fertility, and the knockout lines by CRISPR-Cas9 have larger IM with more florets and set kernels. Further transcriptome analysis showed that expression of auxin, GA, CK, jasmonic acid (JA), and brassinosteroid (BR)-related genes significantly altered between high- and low-ethylene NIL lines, indicating that ethylene may regulate ear development via influencing the phytohormone balance [7].

*BRANCHED SILKLESS1* (*BD1*) encodes an ethylene-responsive element-binding factor (ERF) class TF. In the *bd1* mutant, SMs abnormally produce SPMs, and then SPMs produce SMs repeatedly, which appear like BMs and result in highly branched and reduced fertile florets, indicating that BD1 plays a role in regulating SM identity [186].

#### 3.3.7. The miR172 Pathway and SM Fate Regulation during Maize Ear Development

INDETERMINATE SPIKELET1 (IDS1) and the paralog SISTER OF INDETERMINATE SPIKELET1 (SID1) encode APETALA2 (AP2)-like proteins, the SMs of *ids1* become indeterminate, but the phenotype of *sid1* appears normal [187,188]. The *ids1/sid1* double mutant has fewer KRNs and reduced seed set rates, indicating that IDS1 and SID1 play roles in FM initiation and floral organ functions [188]. In the double mutant, ectopic expression of *AGAMOUS*-like MADS-box genes was observed, indicating that these *AP2* genes act as a negative regulator of *AGAMOUS* in maize [188].

*TASSELSEED4* (TS4) encodes miR172, and *ts4* mutant shows branched ears and irregular floral growth [184]. Further analysis has shown that IDS1 and SID1 are the two main targets of *TS4*, and that *TS4* regulates IDS1 and SID1 at the translation and transcription levels, respectively [184,188]. Interestingly, CG1 mutant plants overexpressing miR156 have lower levels of miR172 [184], indicating that miR172 and miR156 pathways may determine BM formation and FM initiation antagonistically in maize.

#### 3.3.8. Other Genes Regulating IM Activity with an Undetermined Molecular Pathway

Reiterated bifurcation of both the ear and tassel was discovered in a dominant maize mutant *Fascicled ear1* (*Fas1*), and cytological observation of *Fas1* ears showed that development of the central region of the IM has ceased, but growth of the peripheral region continues in the early stage, which results in bifurcation of the ear and increased KRN [189]. Molecular analysis has shown that the duplication and misexpression of a MADS-box gene *MADS8* (*ZMM8*) and the YABBY gene *DROOPING LEAF2* (DRL2) in the *Fas1* locus contributed to the *Fas1* phenotype, and further protein interaction analysis has shown that ZmM8 physically interacted with DRL2 [189].

### 3.4. The Application Potential of the Mined Data for Important Loci Recognition and New Gene Discovery

Three types of data, including the QTL cluster, QTN cluster, and functional genes, were mined in this analysis. It is reasonable to deduce that the common intervals (CIs) that were repeatedly identified by both QTL mapping and GWAS are the most important hotspots regulating ear traits. These CIs can be further divided into two classes. The Class1-CIs are the regions that can be detected simultaneously by QTL mapping and GWAS for specific traits. Only the KRN trait was shortlisted in 19 Class1-CIs, and 5 (26.32%) Class1-CIs possessed cloned genes (Table 1), which further supports the assumption that these Class1-CIs possess crucial functional genes. Therefore, the remaining 11 loci are the direct and important targets for cloning new ear-trait-related genes. We further found that 16 of the 48 (33.33%) cloned genes are located within the chromosomal regions of QTL clusters or QTN clusters (Table S6). The ratios of overlaps between cloned genes and refined clusters are much higher than the previous direct comparisons between associated QTNs and cloned genes [17], indicating that the refined clusters, especially the Class1-CIs, are more instructive for discovering new genes.

The pleiotropic loci have more application potential in breeding, and such loci have been isolated and applied to breeding in maize [190], rice [191], and wheat [192]. To isolate this kind of genetic locus for ear traits (Class2-CIs), we compared all the QTL and QTN clusters and refined 40 Class2-CIs (Table 2). There are 13 Class2-CIs from combined data resources of QTL clusters or QTN clusters, and the remaining 27 Class2-CIs are from single data resources. Six Class2-CIs are related to three types of ear traits, and the remaining 34 Class2-CIs are related to two traits; that is, although the cluster data from eight ear traits are analyzed in combination, the pleiotropic loci that can be repeatedly detected mainly regulate two to three ear traits. Interestingly, only two known genes are found to overlap within the Class2-CIs (Table 2), indicating that most of the candidate genes with pleiotropic effects for ear traits have not been isolated.

We found 16 known genes in 17 QTL clusters (Table S3) and 3 known genes in 2 QTN clusters (Table S5). However, the known genes and summarized molecular networks regulating ear traits are instructive for identifying candidate genes underlying the other QTL or QTN clusters. It is not hard to deduce that if a female IM-expressed gene belonging to a specific pathway is located in such regions, it is more likely to be the candidate or causal gene.

Based on this assumption, we extracted the female IM-expressed genes firstly from published data [26], and then checked the annotation of the genes located in the QTL or QTN clusters. It was found that 71 QTL clusters and 25 QTN clusters contain these kinds of genes (Table S7). More interestingly, some genes belonging to specific pathways are significantly enriched, which provides important potential gene resources for maize ear trait improvement. For example, since auxin is thought to play a central regulatory role in ear traits [193], 10 auxin pathway-related genes residing in seven QTL clusters and two QTN clusters were detected and considered candidate genes for these clusters (Table S7). *IDS1* and *SID1* are two AP2-like protein-encoding genes, which are targets of miR172 [184,188]. Nine AP2 domain-containing protein-encoding genes were identified in eight QTL clusters (Table S7), which are possible targets of miR172 and play roles in regulating maize ear traits. *KRN2* encodes a WD40 protein and knockout *KRN2* in maize, or its ortholog *OsKRN2* in rice, increased grain yield by 10% and 8%, respectively [38]. Nine WD40 genes located in six QTL clusters and three QTN clusters were identified in this study (Table S7), and we suspect that these genes may have potential in increasing maize yield.

## 4. Conclusions and Perspectives

In summary, we refined 153 QTL and 85 QTN clusters, as well as two classes of CIs for ear traits in this study. These loci provide important resources for maize yield improvement by modifying ear traits. At least seven pathways participating in ear trait regulation were refined, and we constructed molecular regulatory networks regulating ear traits based on the known genes. We further predicted the most possible candidate genes for the QTL and QTN clusters depending on the molecular regulatory networks, which provided clues for new gene mining and understanding the molecular basis of ear traits.

Interestingly, 18 known genes are located in the QTL or QTN clusters. Identifying the natural elite alleles of these genes may be more useful for maize breeding. For example, TD1 is a main component of the CLV-WUS pathway, and a mutation of *TD1* severely affects ear development [149]. However, favorable haplotypes derived from natural variation of *TD1* significantly increase KWPE, KNPR, and KRN [193]. In addition, a natural Ser220Asn polymorphism located in an exon of *UB3* was found to be tightly associated with KRN [35]. We thus think it is worthwhile to test the correlation of the natural variations of other known genes located in the QTL clusters or QTN clusters with ear traits.

In addition to natural variation, the creation of weak alleles in the core genes is another strategy for improving ear traits, and many successful examples have been reported. For example, weak alleles with a partial loss of FEA2 function were generated by ethyl methanesulfonate (EMS)-induced mutagenesis, and the weak alleles can enhance IM size and KRN without reducing EL or causing fasciation [152]. A similar phenomenon was also observed in *FEA3*, which locates in a QTL cluster for KRN (Table S5). By comparison with *FEA2*, the weak alleles of *FEA3* increased both the ear traits and the overall yield [151]. CRISPR–Cas9 genome-editing technology developed quickly and has been successfully applied in maize genome research [194,195,196]. The weak promoter alleles of CLE genes, including *ZmCLE7*, were newly developed by using CRISPR–Cas9 genome-editing technology, and the ear development of these gene editing lines was relatively normal, with grain yields per ear increasing from approximately 14% to 26% compared with WT [197].

However, only a few ear-trait-related genes have been cloned, which has impeded the further improvement of ear traits. The predicted candidate genes deserve to be quickly verified, considering that many of them belong to known pathways (Table S6). With the accumulation of information from both genetic and molecular mechanisms, synergetic improvement of ear traits by beneficial QTL pyramiding is possible. We propose that combining elite allele-generating technologies such as CRISPR–Cas9 genome editing with marker-assisted selection (MAS) or genomic selection (GS) will speed up progress in plant breeding. For example, the direct knockout of ear trait genes (such as *KRN2*) and the generation of quantitatively variated alleles benefiting breeding (such as *ZmCLE7*) have been reported, and high-throughput CRISPR/Cas9 mutagenesis has recently been developed [38,197]. In addition, both traditional MAS and modern GS technologies have also been applied for crop improvement [198,199]. In maize, the utilization of favorable haplotypes of *TD1* aided by MAS was proposed to be more efficient than the standard maize breeding procedures [193]. We thus believe that combining technologies for the high-throughput creation of new variations of the candidate genes with the high-efficiency selection of elite alleles should be effective strategies for maize yield improvement by modifying the ear traits.

## Figures and Tables

**Table 1:** Summary of the common intervals that can be detected simultaneously by QTL mapping and GWAS.

Number	Chromosome	QTL Cluster		QTN Cluster		Known Gene
		Start (Mb)	End (Mb)	Start (Mb)	End (Mb)	
1	1	293.44	304.60	297.63	298.13	
2	1	293.44	304.60	299.78	300.29	*IDS1/KRN1*
3	2	5.47	5.85	4.84	5.83	
4	2	19.86	26.25	21.48	22.02	
5	2	19.86	26.25	23.49	24.19	
6	3	14.89	26.40	17.27	18.08	
7	3	226.09	228.09	225.85	226.54	
8	4	195.32	239.24	204.74	205.67	*RKN4, UB3*
9	4	195.32	239.24	205.76	206.52	*RKN4, UB3*
10	5	20.41	23.71	22.80	23.30	
11		36.01	51.50	35.58	36.47	
12	5	36.01	51.50	41.07	41.62	
13	5	36.01	51.50	44.21	44.71	
14	5	60.99	67.09	65.79	66.29	*TD1*
15	5	141.98	184.11	146.17	146.67	
16	7	11.68	12.63	12.00	12.62	
17	7	21.96	45.32	41.96	42.46	*SID1*
18	8	112.72	122.82	113.29	113.98	
19	9	27.76	29.76	28.29	29.03	

Abbreviations: Mb, mega-base pairs; QTL, quantitative trait locus; QTN, quantitative trait nucleotide.

**Table 2:** Summary of the pleiotropic loci for ear traits.

No.	Chromosome	Pleiotropic Region		Data Resource	Ear Traits	Gene
		Start (Mb)	End (Mb)			
1	1	0.00	0.61	QTN cluster	CD, KRN	
2	1	1.96	2.11	QTN cluster	KRN, ED, CD	
3	1	2.50	2.68	QTN cluster	CD, KRN	
4	1	3.23	3.72	QTN cluster	CD, ED	
5	1	26.40	26.90	QTN cluster	EL, KRN	
6	1	28.45	28.48	QTN cluster	EL, CD, KRN	
7	1	34.89	35.55	QTN cluster/QTL cluster	CD, EL	
8	1	35.99	36.49	QTN cluster/QTL cluster	EL, ED	
9	1	42.54	43.54	QTL cluster	EL, KNPR	
10	1	44.18	44.90	QTL cluster	KWPE, ED	
11	1	220.28	221.93	QTL cluster	KRN, CD	
12	1	285.34	292.19	QTL cluster	KRN, KWPE	
13	2	23.49	23.81	QTN cluster	CD, KRN	
14	2	65.94	66.23	QTL cluster	KRN, KNPR	*BA2*
15	2	153.51	154.97	QTL cluster	EL, KRN	
16	2	200.01	200.07	QTL cluster	KRN, ED	
17	3	2.42	3.42	QTN cluster/QTL cluster	CD, KRN	
18	3	3.19	3.28	QTL cluster	CD, KRN, EL	
19	3	9.57	10.06	QTL cluster	EL, KRN	
20	3	10.44	11.10	QTL cluster	KRN, EL	
21	3	24.90	25.40	QTN cluster/QTL cluster	KRN, CD	
22	3	154.92	155.42	QTN cluster/QTL cluster	KRN, ED	
23	4	0.33	0.83	QTN cluster	CD, KRN	
24	4	191.57	191.82	QTN cluster/QTL cluster	KRN, ED, EW	
25	4	215.58	216.26	QTN cluster/QTL cluster	KRN, EL	
26	4	244.05	244.61	QTN cluster/QTL cluster	KRN, ED	
27	4	244.74	245.54	QTL cluster	KRN, ED	
28	5	126.98	127.53	QTN cluster/QTL cluster	EL, KRN	
29	5	137.90	138.57	QTL cluster	KNPR, EL	
30	5	145.16	145.22	QTL cluster	CW, KRN, KWPE	
31	5	164.69	166.10	QTL cluster	KRN, EL	
32	5	196.69	197.81	QTL cluster	EL, KRN	
33	5	213.77	214.96	QTL cluster	KRN, EL	
34	5	216.13	216.72	QTN cluster/QTL cluster	EL, CD	
35	8	121.24	121.27	QTN cluster/QTL cluster	KRN, CD, EL	
36	8	167.11	167.61	QTN cluster/QTL cluster	KRN, ED	
37	9	10.90	12.21	QTL cluster	ED, KRN	
38	9	13.09	14.78	QTL cluster	ED, KRN	
39	10	94.76	98.77	QTL cluster	EW, KNPR	*ACO2*
40	10	148.31	148.42	QTN cluster/QTL cluster	KRN, EL	

Abbreviations: CD, cob diameter; CW, cob weight; ED, diameter; EL, ear length; EW, ear weight; KRN, kernel row number; KNPR, kernel number per row; KWPE, kernel weight per ear; Mb, mega-base pairs; QTL, quantitative trait locus; QTN, quantitative trait nucleotide.

## Data Availability

All data are shown in the main manuscript and in the Supplementary Materials.

## Supplementary Materials

The following supporting information can be downloaded at: https://www.mdpi.com/article/10.3390/cells12141900/s1, Table S1: The list of explanations of abbreviations that appeared in this study; Table S2: Information on the normalized original QTL data collected in this study; Table S3: Summary of the information on QTL clusters; Table S4: Information of the normalized original QTN data collected in this study; Table S5: Summary of the information on QTN clusters; Table S6: Summary of the information on cloned ear trait genes; Table S7: Information on the predicted candidate genes for the QTL and QTN clusters.
